# Towards a Phylogenomic Framework for the *Fusarium oxysporum* Species Complex

**DOI:** 10.3390/ijms27146255

**Published:** 2026-07-14

**Authors:** Juliana Lopez-Jimenez, Juan M. Daza, Juan F. Alzate

**Affiliations:** 1 Centro Nacional de Secuenciación Genómica—CNSG, Sede de Investigación Universitaria—SIU, Universidad de Antioquia UdeA, Calle 70 No. 52-21, Medellin 050010, Colombia; extension.cnsg@udea.edu.co; 2 Grupo Herpetológico de Antioquia GHA, Instituto de Biología, Facultad de Ciencias Exactas y Naturales, Universidad de Antioquia UdeA, Calle 70 No. 52-21, Medellin 050010, Colombia; juanm.daza@udea.edu.co; 3 Departamento de Microbiología y Parasitología, Facultad de Medicina, Universidad de Antioquia UdeA, Calle 70 No. 52-21, Medellin 050010, Colombia

**Keywords:** *Fusarium oxysporum* species complex, Phylogenomics, Taxonomy, Genomic diversity, *Fusarium fabacearum*, *Fusarium cugenangense*, *Fusarium odoratissimum*

## Abstract

The *Fusarium oxysporum* species complex (FOSC) is a genetically diverse and globally distributed group of fungi that includes both pathogenic and non-pathogenic lineages with broad host ranges and major agricultural importance. To provide a higher-resolution view of its diversity and evolutionary history, we assembled and quality-filtered genomes, generating a curated dataset of 336 high-quality assemblies complemented by seven NCBI reference genomes (total 343). Orthology inference recovered 4286 conserved single-copy coding sequences, which resolved evolutionary relationships across the complex with high confidence. This framework recognizes three major clades, resolves several taxonomic inconsistencies, and delineates phylogenomic boundaries among distinct lineages. Despite overall nucleotide identities exceeding 96%, whole-genome comparisons consistently supported the existence of distinct species lineages. Within Clade 2, we identified two previously unrecognized lineages: the *Fusarium* “afroindicum” clade, distributed across Africa and Asia, and the *Fusarium* “europaeum” clade, restricted to Europe. Both lineages are genetically coherent, ecologically distinct, and strongly supported by phylogenomic evidence. Finally, a global metadata survey revealed that *Fusarium fabacearum*—rather than *F. oxysporum* sensu stricto—is the most widespread and host-diverse member of the complex. Collectively, this large-scale assembly effort and comprehensive phylogenomic framework provide the most robust evolutionary context to date for the FOSC and shed light on its global diversity and ecological breadth.

## 1. Introduction

The *Fusarium oxysporum* species complex (FOSC) is a widely spread group of fungi that significantly impacts agriculture and has well-documented effects on human and animal health [1,2,3]. Several species within this complex are common around the world and pose a persistent and growing threat to global agriculture due to their ability to infect a very broad range of plant hosts [4,5] while the complex also includes non-pathogenic strains [6,7,8,9].

Members of the *Fusarium oxysporum* species complex (FOSC) are causal agents of root rot and wilt diseases that affect a broad spectrum of economically important crops. Reported hosts span fruits such as strawberries, bananas, and date palms; vegetables including tomatoes, cucumbers, lettuce, and coriander; legumes such as chickpeas and yams; and industrial or specialty plants including cotton, hemp, coffee, and ginger. This remarkable host range has been documented across multiple continents in a wide body of studies [10,11,12,13,14,15,16,17,18,19,20,21,22,23,24,25,26,27,28,29,30,31,32,33,34,35].

Recognized as one of the most economically damaging groups of plant pathogens worldwide [36], FOSC is consistently linked to significant yield losses and long-term soil persistence [4,37], making its eradication and management especially difficult [38,39]. Nonetheless, certain *F. oxysporum* strains have been described as saprophytic, meaning they are capable of growing on dead or decaying organic matter, although they can also act as pathogens under certain conditions [40,41], highlighting the importance of a reliable, high-resolution, and high-precision taxonomic framework that enables the identification and tracking of different species or even strains within the complex.

The current definition of *Fusarium oxysporum* remains broad and somewhat ambiguous [42], as it depends heavily on morphological traits and phenotypic subspecific classifications—such as *formae speciales*, races, and vegetative compatibility groups (VCGs). Additionally, species historically categorized within the FOSC are notoriously difficult to identify based solely on microscopic morphology or culture traits. Diagnostic features, such as conidial size and shape, septation patterns, or the arrangement of phialides, often overlap significantly among different taxa, and culture-based characteristics can vary depending on growth conditions and media composition [43]. As a result, identifications can vary significantly across laboratories and often depend on the individual expertise of the analyst, which limits the reproducibility and reliability of purely morphological methods. Historically, host-based classification systems, particularly the *formae speciales* framework, have been used to group isolates by host range and further subdivide them into races based on their virulence spectra. While this provided a useful historical scheme, its limitations have become more apparent, as they fail to capture the underlying evolutionary relationships [6,12,44].

In response to the limitations of host-based classifications and purely morphological approaches, molecular methods began to emerge in the mid-90s, initially focusing on PCR amplification and single-locus sequencing of ribosomal DNA regions [45]. While rDNA became the universal fungal barcode, it proved to have very limited resolution. With the advent of advanced sequencing technologies, additional molecular markers were incorporated into FOSC research, introducing multilocus sequence analysis (MLSA), which primarily relied on phylogenetic methods. MLSA schemes expanded to include dozens of markers, improving upon rDNA resolution; however, issues such as paralogy, recombination, insufficient phylogenetic resolution, and the lack of standardized marker sets often produced incongruent topologies and restricted cross-study comparability. Nonetheless, loci such as translation elongation factor 1α (*tef1*), RNA polymerase II second largest subunit (*rpb2*), β-tubulin (*tub2*), and calmodulin A (*cmdA*) played a pivotal role in shaping early species delimitation hypotheses within the FOSC [42,46,47,48,49,50,51,52].

Genome-wide phylogenomic approaches have become essential for resolving the long-standing taxonomic issues of the FOSC. Members of the FOSC typically exhibit genome sizes ranging from approximately 45 to 70 Mb, although substantial variation exists among lineages due to differences in accessory genomic content [53]. This variation in genomic content is primarily driven by horizontal gene transfer (HGT) of effector genes between lineages, contributing to the observed differences in pathogenicity and adaptability. These effector genes, often located on accessory chromosomes, play a crucial role in virulence and adaptation to different host plants, enabling different lineages to acquire traits that enhance their pathogenic potential [54]. Recent research using large-scale datasets of conserved single-copy genes has offered unprecedented insights into its evolutionary history. A core-genome phylogenomic framework that integrates hundreds of loci revealed genome-wide evolutionary patterns, such as GC content variation and lineage-specific duplication events, thereby refining our understanding of genomic diversity and adaptation within the genus [55]. Extending this framework, a core-gene phylogeny based on 3811 conserved single-copy BUSCO genes from 69 banana-infecting strains and 55 additional isolates infecting other hosts demonstrated that banana pathogenic forms are polyphyletic, distributed across multiple major clades of the FOSC, underscoring that pathogenicity toward banana has arisen multiple times independently [56]. Complementary population genomics based on 410 assembled genomes revealed a predominantly clonal evolutionary pattern, with divergence dating suggesting that major lineages separated approximately 500,000 years ago. By integrating phylogenomic concordance with population-level signals of recombination, this study provided one of the most comprehensive resources for the FOSC and reinforced the recognition of well-supported phylogroups as species-level lineages [57]. High-quality genome assemblies, like those of *F. oxysporum* QK8, enabled strong phylogenomic analysis across 24 other *Fusarium* species, showing the power of whole-genome sequencing to link genome structure, gene family increases, and pathogenicity factors to evolutionary change [20]. Similarly, effector-based comparative genomics has shown that host-specific effector repertoires may transcend phylogenetic boundaries, highlighting their importance in the independent emergence of pathogenic lineages within the FOSC. These patterns are consistent with a significant contribution of horizontal gene transfer of effector genes, often involving accessory chromosomes [58].

With this work, our goal was to create a curated and reproducible phylogenomic framework to address key questions about the evolutionary history of the FOSC, clarify relationships among its recognized species, and test its monophyly. Additionally, we aimed to offer insights into the evolution of overall genomic features within the complex, enabling the integration of geographical distributions and host associations, and allowing long-standing paradigms to be contrasted with evidence from modern high-resolution technologies.

## 2. Results

### 2.1. Genome Statistics and Quality Assessment

We compiled a comprehensive collection of publicly available sequencing datasets for members of the *Fusarium oxysporum* species complex (FOSC) annotated as *Fusarium oxysporum*, *Fusarium foetens*, *Fusarium veterinarium*, or *Fusarium odoratissimum*. Application of these selection criteria resulted in the retention of 721 SRA entries.

All sequencing datasets were cleaned and de novo assembled as described in Section 4. To ensure high-quality assemblies, we evaluated several parameters, including total assembly size, number of scaffolds, scaffold N50, average sequencing depth, number of alternate alleles, GC content, proportion of ambiguous bases (Ns), and BUSCO-based genome quality metrics (completeness and proportion of duplicated genes).

Descriptive statistics revealed the presence of low-quality assemblies, including one with a total size of <1 Mb and several others below 40 Mb. Conversely, some assemblies showed evidence of possible contamination, with total lengths exceeding 80 Mb, and in extreme cases >100 Mb. Additional issues included highly fragmented assemblies with more than 100,000 scaffolds and very low contiguity (N50 < 1000 bp).

To filter out such poor-quality assemblies, we applied the following thresholds: N50 > 50,000 bp, assembly size between 44 Mb and 75 Mb, a maximum of 50,000 scaffolds, and a minimum sequencing depth of 10×. Although some of these thresholds were intentionally permissive, only about half of the assemblies passed the quality filters (*n* = 336).

To assess the presence and extent of potential library contamination by non-target organisms, raw sequencing reads were taxonomically classified using Kraken2 and Bracken. Although these tools do not provide definitive organismal identification, they are effective for identifying contaminated libraries and for broadly inferring the likely biological origin of non-target sequences. Across the initial dataset of 721 genome assemblies, substantial contamination was observed in a subset of read sets; however, after applying quality-control and filtering criteria, contamination was markedly reduced, with a median of 97% of reads assigned to the genus *Fusarium* in the retained SRA accessions. Only three samples exhibited a dominant taxon other than *Fusarium* at the read level, all of which were bacterial, specifically *Stenotrophomonas* (72%), *Serratia* (77%), and *Achromobacter* (52%). Plant-derived sequences were rare and detected in only five datasets, with reads assigned to *Asarum* in two samples (13.7% and 5.42%) and to *Lasthenia* in three samples (0.77–1.7%).

The most frequently detected secondary genera across the dataset included *Timema* (103 samples), Betacoronavirus (52), *Ilyonectria* (43), *Pseudomonas* (31), *Staphylococcus* (29), *Terrapene* (10), Nectriaceae (7), *Achromobacter* (7), *Ralstonia* (6), and *Homo* (6). These assignments span a wide range of biological origins and are best interpreted in the context of metagenomic classification uncertainty combined with the heterogeneous provenance of publicly available sequencing datasets.

Given this signal of residual contamination, an additional filtering step was applied to minimize potential bias in genome assembly size and GC content estimates. This step involved BLASTN v2.12.0+ alignments of the 336 retained assemblies against a curated reference database comprising representative FOSC genomes with the lowest contamination signals. Median genome assembly size and GC content were then compared per species before and after filtering. GC content was minimally affected, with changes ranging from 0% to 0.12%, indicating that residual non-*Fusarium* sequences had only a marginal influence on species-level median GC estimates. In contrast, genome assembly sizes showed a modest but expected reduction after filtering, with a median decrease of approximately 3%. Reductions ranged from 0.25% in *Fusarium pharetrum* to 10.1% in *Fusarium* sp. FOSC-G4_3 (*Fusarium* “afroindicum”). For *Fusarium fabacearum*, the species with the largest representation in the dataset, the reduction in assembly size was consistent with this overall trend (6.75%).

As an additional measure of genome purity and clonality, all raw reads were mapped back to their respective assemblies, and the number of alternate alleles was quantified. The resulting distributions showed considerable variation: whereas many assemblies contained only a few hundred alternate alleles, consistent with highly clonal genomes, a small subset exceeded 50,000, indicative of substantial intra-sample heterogeneity. Assembly sizes in the FOSC filtered genome assemblies ranged from 45.5 Mb to 57.6 Mb, with a median of 52 Mb, and the GC content showed a median value of 47.5%, with a range between 46.8% and 48.5%. These values are consistent with the expected genome range for FOSC members, indicating that all filtered assemblies fall within expected limits.

**Figure 1:**
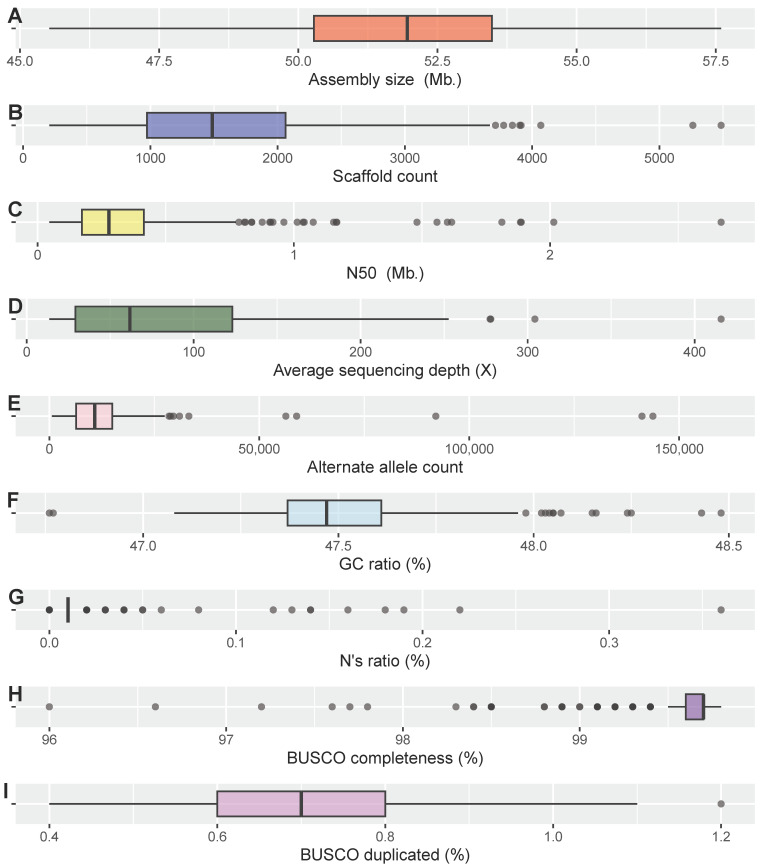
Assembly quality metrics for *Fusarium oxysporum* species complex (FOSC) genomes. Boxplots summarize the distribution of nine genomic features across the quality-filtered FOSC dataset: (**A**) assembly size (Mb), (**B**) scaffold count, (**C**) N50 (Mb), (**D**) average sequencing depth (×), (**E**) alternate allele count, (**F**) GC content (%), (**G**) proportion of ambiguous bases (N’s ratio, %), (**H**) BUSCO completeness (%), and (**I**) BUSCO duplication rate (%). Points represent outliers beyond the whiskers of each boxplot.

Across the 336 filtered genome assemblies, the number of scaffolds ranged from 206 to 5485, with a median of 1486. The assembly contiguity, measured by N50, had a median value of 278,470 bp, with values reaching up to 2.67 Mb. The proportion of ambiguous bases (N ratio) was generally low, with a median of 0.01% and a maximum of 0.36%. Sequencing depth across assemblies had a median of 61.8×, ranging from 13.6× to 416×. Regarding clonality, the number of alternate alleles per genome varied widely, ranging from 596 to 143,753, with a median of 10,795. Finally, completeness assessed by BUSCO was consistently high, with values between 96% and 99.8%, and a median of 99.7% (Figure 1).

In addition, seven publicly available reference assemblies of the same complex were downloaded from NCBI, resulting in a final dataset of 343 genomes for phylogenomic analysis.

### 2.2. Phylogenomic Reconstructions

A phylogenomic analysis was conducted using 343 taxa and 4286 single-copy ortholog CDSs, resulting in an alignment of 6,998,306 nucleotide sites with only 0.0012% missing data. Of these, 94.1% (6,586,950 sites) were constant, while 315,389 positions (4.5%) were parsimony informative, corresponding to 166,867 distinct site patterns. Model testing selected an Invar+FreeRate model with six categories, estimating that 62.3% of sites were invariant and that the remaining variable sites were distributed across a small proportion of rapidly evolving positions. The inferred maximum-likelihood tree had a log-likelihood of −16,094,992, with a total branch length of 0.1334, of which 71.8% corresponded to internal branches. Although overall branch support was high, IQ-TREE flagged 43 near-zero internal branches (<0.0001), suggesting that certain relationships remain poorly resolved due to the high sequence similarity among genomes at terminal nodes, all of which occurred within species-level clades. The outgroup *Fusarium foetens* was included for rooting purposes.

The topology of the phylogenomic tree clearly resolved, with high confidence, the relationships among the basal nodes relative to the currently accepted *Fusarium* species. Within the FOSC, three main clades were recovered. Clade 1 (*F. odoratissimum*) represents the most basal lineage of the complex, followed by two large and diverse clades designated as Clades 2 and 3, following the proposal initially described by van Westerhoven et al. [56].

**Figure 2:**
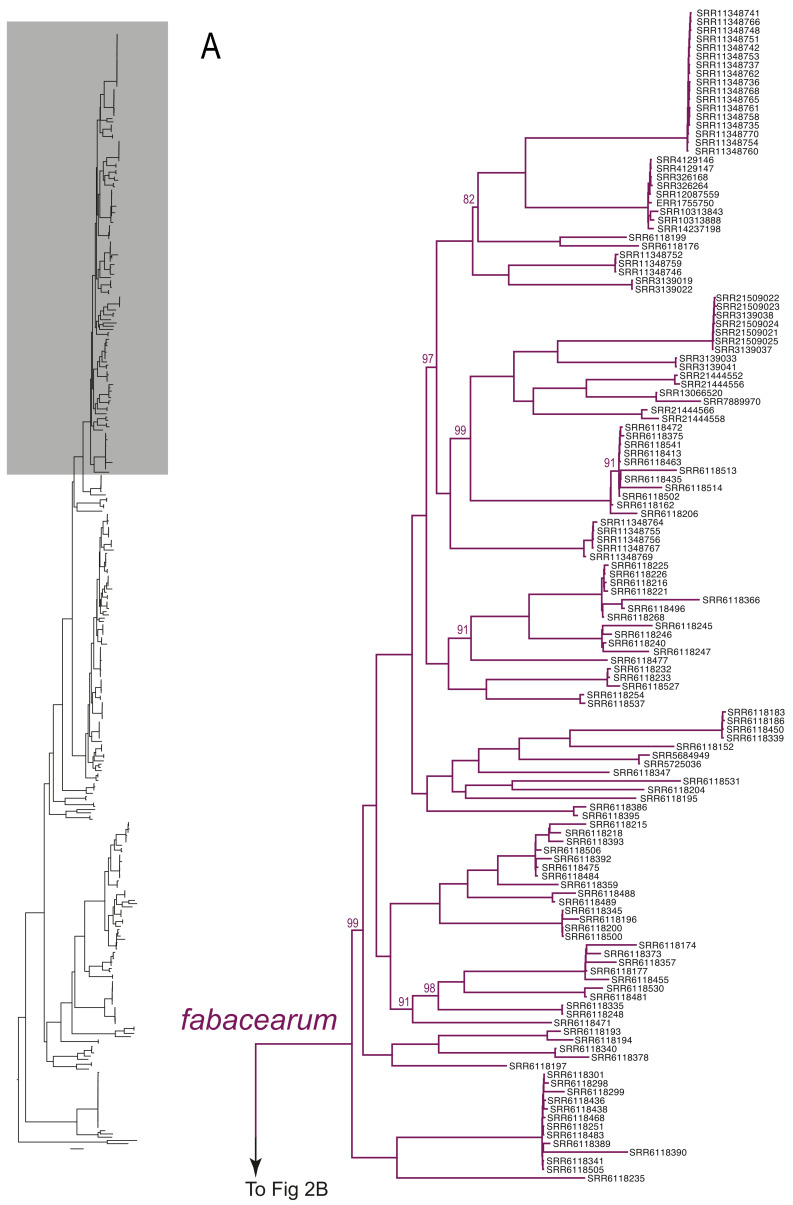

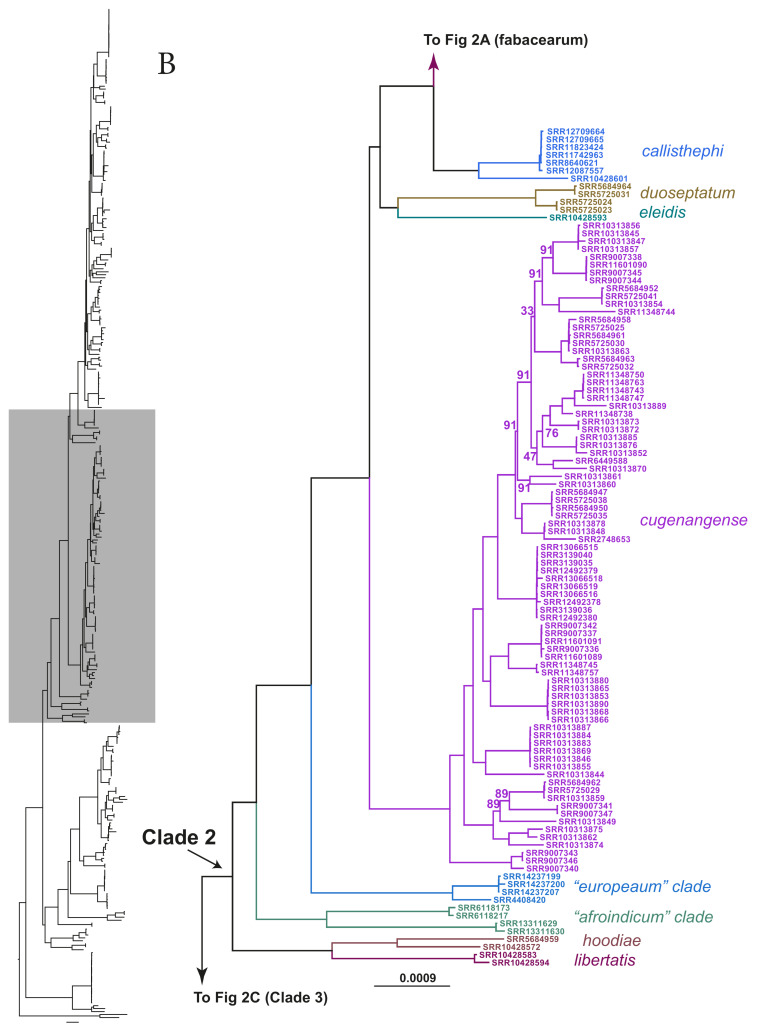

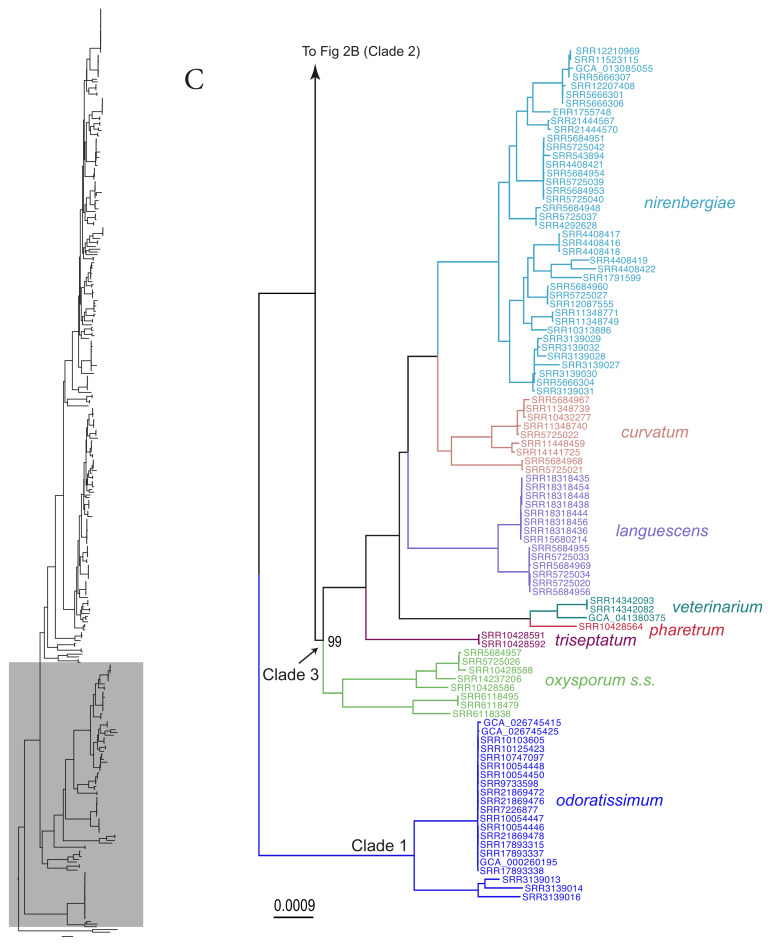
(**A**). Maximum-likelihood phylogenomic tree of the *Fusarium oxysporum* species complex. Global phylogeny inferred with IQ-TREE from 4286 concatenated single-copy orthologous coding sequences (CDSs). Ultrafast bootstrap (UFB) support values are displayed at nodes (only values <100 are indicated). Terminal branches were collapsed where multiple genomes of the same monophyletic lineage were present. The three major FOSC clades are highlighted in distinct colors, with alternative lineage nomenclatures provided to facilitate systematic cross-comparison. (**B**). Detailed phylogenomic relationships within FOSC Clade 2. Continuation of the maximum-likelihood tree framework from (**A**), highlighting high-resolution evolutionary relationships within Clade 2. Ultrafast bootstrap (UFB) support values <100 are shown at nodes, and distinct species and provisional lineages are distinguished by specific colors. (**C**). Detailed phylogenomic relationships within FOSC Clade 3. Continuation of the maximum-likelihood tree framework from (**A**,**B**), showing high-resolution evolutionary relationships within Clade 3. Ultrafast bootstrap (UFB) support values <100 are shown at nodes, and discrete lineages are color-coded.

Clade 2 encompasses *F. libertatis*, *F. hoodiae*, *F. cugenangense*, *F. elaeidis*, *F. duoseptatum*, *F. callistephi*, and *F. fabacearum*. Notably, two well-supported clades, not previously assigned names, were recovered as sisters to the lineage comprising *F. cugenangense* + *F. elaeidis* + *F. duoseptatum* + *F. callistephi* + *F. fabacearum*. These two lineages, provisionally designated as the *Fusarium* “afroindicum” and *Fusarium* “europaeum” clades, show consistent placement and strong phylogenomic support, justifying their recognition as distinct lineages within Clade 2 of the FOSC. Figure 2A shows the maximum-likelihood phylogenomic tree of the *Fusarium oxysporum* species complex, with *F. fabacearum* being the most represented species in Clade 2. Figure 2B depicts the rest of the species within Clade 2, including *F. callistephi*, *F. duoseptatum*, *F. elaeidis*, *F. cugenangense*, the “europaeum” and “afroindicum” clades, *F. hoodiae*, and *F. libertatis*. Figure 2C shows Clade 1 (*F. odoratissimum*) and Clade 3, which contains other species such as *F. oxysporum* s. str., *F. triseptatum*, *F. pharetrum*, *F. veterinarium*, *F. languescens*, *F. curvatum*, and *F. nirenbergiae*. Clade 2 contained most of the analyzed genomes (243 assemblies), followed by Clade 3 (78 assemblies) and Clade 1 (21 assemblies). The complete list of species assignments obtained with our phylogenomic approach, compared with the original species assignments reported in the NCBI SRA, is provided in Supplementary Table S2.

**Figure 3:**
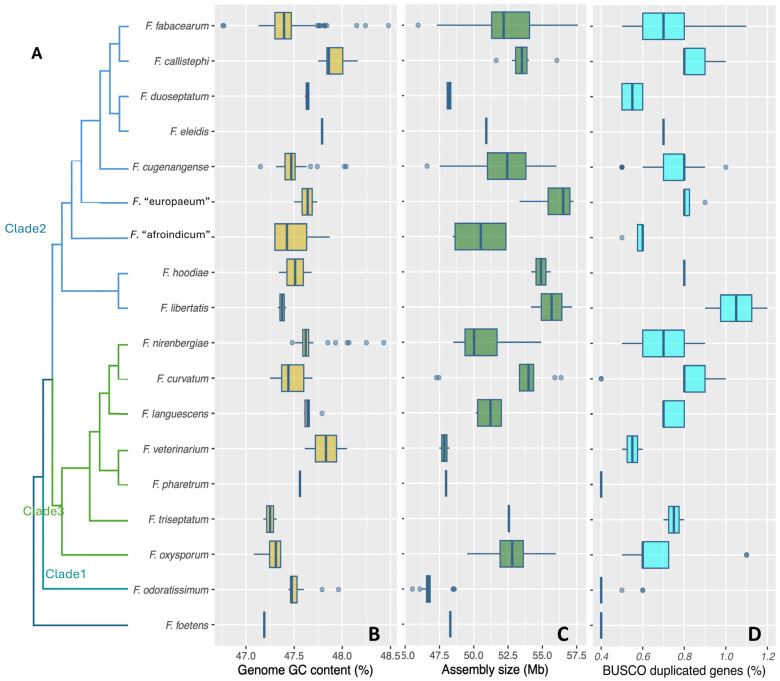
Comparative global genome properties across *Fusarium oxysporum* species complex (FOSC) clades. (**A**) Reference cladogram illustrating the evolutionary architecture of the primary FOSC clades and provisional lineages. (**B**) Distribution of genome GC content (%) across individual lineages. (**C**) Distribution of genome assembly sizes (Mb) across species. (**D**) Relative percentages of conserved, single-copy, and duplicated BUSCO genes across major clades.

Having resolved with confidence the evolutionary history of the FOSC, we next examined the evolution of genome size and GC content. Median genome size varied across species. The most significant number of genomes corresponded to *F. fabacearum* (136 assemblies; median 52.2 Mb) and *F. cugenangense* (83 assemblies; median 52.4 Mb). Other well-represented lineages included *F. nirenbergiae* (40 assemblies; median 50 Mb) and *F. languescens* (15 assemblies; median 51.2 Mb). Additional representatives were *F. odoratissimum* (21 assemblies; median 46.7 Mb), *F. curvatum* (9 assemblies; median 54 Mb), *F. callistephi* (7 assemblies; median 53.5 Mb), *F. oxysporum* s. str. (8 assemblies; median 52.8 Mb), and *F. duoseptatum* (4 assemblies; median 48.2 Mb). The two novel, provisionally designated lineages within Clade 2—*Fusarium* “afroindicum” and *Fusarium* “europaeum”—each comprised four assemblies, with median assembly sizes of 50.5 Mb and 56.5 Mb, respectively. Genome GC content showed subtle but consistent variation across groups, with medians ranging from ~47.2% to ~47.9%. Core genome duplication events, measured by the BUSCO duplicated genes index, displayed a median value of 0.7% across all FOSC genomes analyzed (Figure 3).

**Figure 4:**
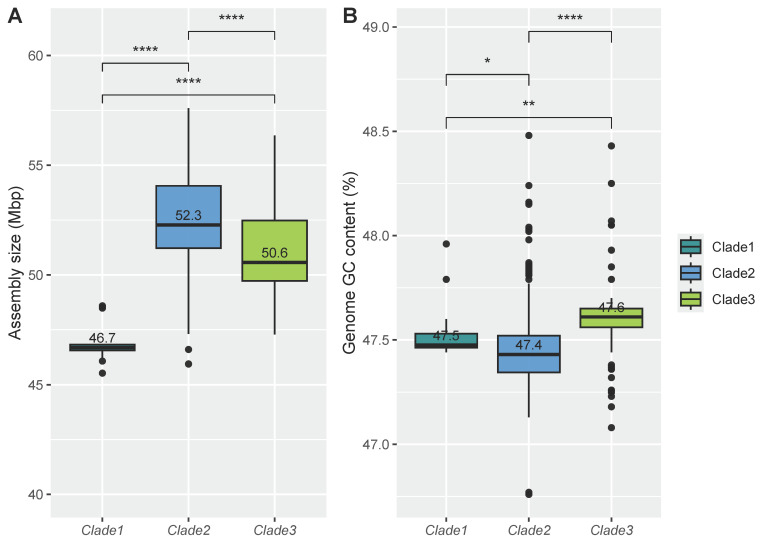
Comparative distribution of global genome properties among primary FOSC clades. (**A**) Genome assembly size (Mbp) variations across the three main clades. (**B**) Whole-genome GC content (%) distributions. Median values are displayed numerically above each boxplot. Significant differences between clades were calculated using pairwise Wilcoxon rank-sum tests with Benjamini–Hochberg multiple-testing corrections (ns = not significant; * = *p* ≤ 0.05; ** = *p* ≤ 0.01; **** = *p* ≤ 0.0001).

Variation in genome size and GC content occurred largely independently across species, reinforcing the concept of high genomic plasticity of these fungi. However, a clear tendency for FOSC clade 2 to harbor larger genomes can be observed. To confirm this, we grouped all assemblies according to their FOSC clade and compared median genome sizes and GC content. The results supported the visual observation: Clade 2 displayed the largest genomes, with a median size of 52.3 Mb, while Clade 3 genomes were smaller (median 50.6 Mb), a difference of 1.7 Mb. Clade 1 contained the most compact genomes, with a median around 46.7 Mb. Statistical tests confirmed these differences, with *p*-values < 0.0001. A similar comparison was performed for GC content. Here, differences were more subtle but still significant, with Clade 3 exhibiting slightly higher GC levels than Clade 2, corresponding to a median difference of ~0.2% (Figure 4).

**Figure 5:**
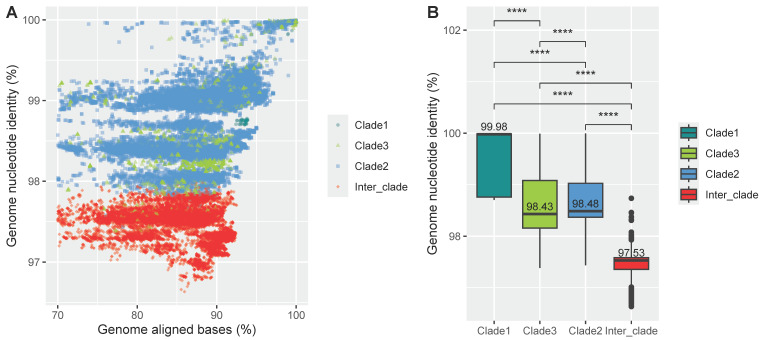
Whole-genome sequence conservation and core identity boundaries among main FOSC clades. (**A**) Pairwise whole-genome comparisons illustrating the relationship between the proportion of aligned bases (%, *x*-axis) and average nucleotide identity (ANI, %, *y*-axis) across all analyzed FOSC genomes. Data points are partitioned into intra-clade and inter-clade (Inter_clade) comparisons, with coordinates colored by lineage. (**B**) Boxplots displaying the distribution of genome average nucleotide identity values (%) within individual clades versus between-clade pairings (INTER_clade). Median values are indicated above each box. Pairwise statistical differences were assessed using the Wilcoxon rank-sum test (**** = *p* ≤ 0.0001).

To assess genome-wide conservation among FOSC, we performed pairwise genome alignments. We applied this approach by segregating the FOSC into the three main clades and comparing genomes both within clade (intra-clade) and between clades (inter-clade). Overall, most genomes exhibited at least 70% alignment coverage, with nucleotide identity of aligned blocks ranging from over 96% to nearly 100%. As expected, the median nucleotide identity between FOSC clades (inter-clade) was significantly lower (97.53%, *p* < 0.0001) compared to within-clade values, which reached 99.9% for clade 1 (*F. odoratissimum*), 98.43% for clade 3, and 98.48% for clade 2 (Figure 5).

**Figure 6:**
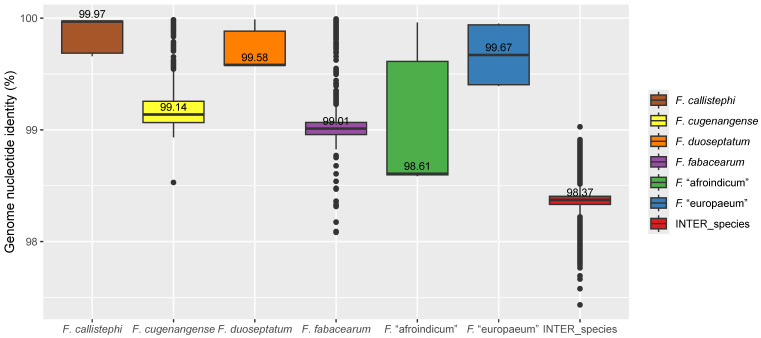
Interspecific and intraspecific genome nucleotide identity within FOSC Clade 2. Boxplots illustrate whole-genome average nucleotide identity (ANI) values (%, *y*-axis) calculated within individual species/lineages and across cross-species comparisons (INTER_species) within Clade 2. Horizontal bars and numerical annotations represent median values. Statistical outliers are indicated by black points.

The FOSC Clade 2 contains most of the genomes classified within the complex and appears to be the most diverse, comprising seven accepted species and two additional, well-supported and novel genomic lineages. We therefore analyzed genome conservation and nucleotide identity within this clade in greater detail. Within each species, as well as within the provisional *Fusarium* “afroindicum” and *Fusarium* “europaeum” clades, the median nucleotide identity was above 99%, except for the “afroindicum” lineage, which showed a slightly lower value of 98.61%. By contrast, inter-species comparisons within clade 2 yielded lower identities, with a median of 98.37% (Figure 6).

**Figure 7:**
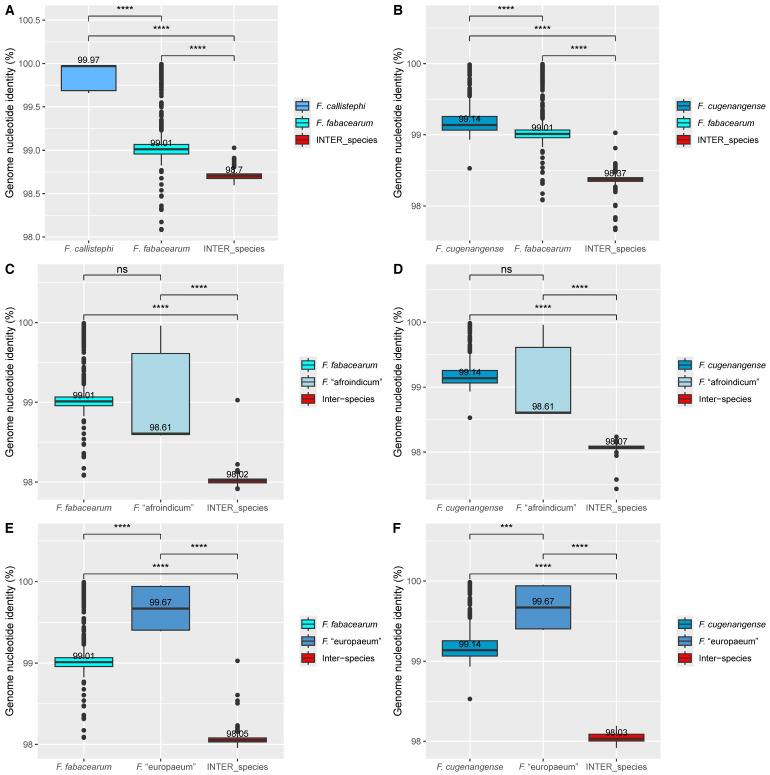
Pairwise genomic identity boundaries among distinct FOSC Clade 2 lineages. Boxplots display genome-wide nucleotide identity ranges (%) computed from pairwise comparisons between selected Clade 2 reference taxa and provisional lineages: (**A**) *F. fabacearum* vs. *F. callistephi*, (**B**) *F. fabacearum* vs. *F. cugenangense*, (**C**) *F. fabacearum* vs. provisional lineage *Fusarium* “afroindicum, (**D**) *F. cugenangense* vs. provisional lineage *Fusarium* “afroindicum”, (**E**) *F. fabacearum* vs. provisional lineage *Fusarium* “europaeum”, and (**F**) *F. cugenangense* vs. provisional lineage *Fusarium* ”europaeum”. Medians are displayed directly above each boxplot. Statistical differences were evaluated using pairwise Wilcoxon rank-sum tests (ns = not significant; *** = *p* ≤ 0.001; **** = *p* ≤ 0.0001).

Between the sister species *F. fabacearum* and *F. callistephi*, the median nucleotide identity was 98.7%, increasing to 99.01% within (intra-species) *F. fabacearum* and 99.97% within *F. callistephi*. A comparable trend was observed when *F. fabacearum* was compared with *F. cugenangense*, the species with the largest genomic representation: the inter-species median nucleotide identity was 98.37%, while within *F. cugenangense* it reached 99.14%. The lineages provisionally designated as *Fusarium* “afroindicum” and *Fusarium* “europaeum” exhibited inter-species nucleotide identities of 98.02% and 98.05%, respectively, when compared with *F. fabacearum*, and 98.07% and 98.03% against *F. cugenangense*, respectively (Figure 7A–F). Consistently, phylogenetic distances estimated supported these relationships, showing a distance of 0.001 between *F. fabacearum* and *F. callistephi*, 0.0014 between *Fusarium* “afroindicum” and the common ancestor of *Fusarium* “europaeum” + *F. fabacearum*, and 0.0024 between *Fusarium* “europaeum” and the ancestor of *F. fabacearum* + *F. cugenangense* (Supplementary Figure S1).

**Figure 8:**
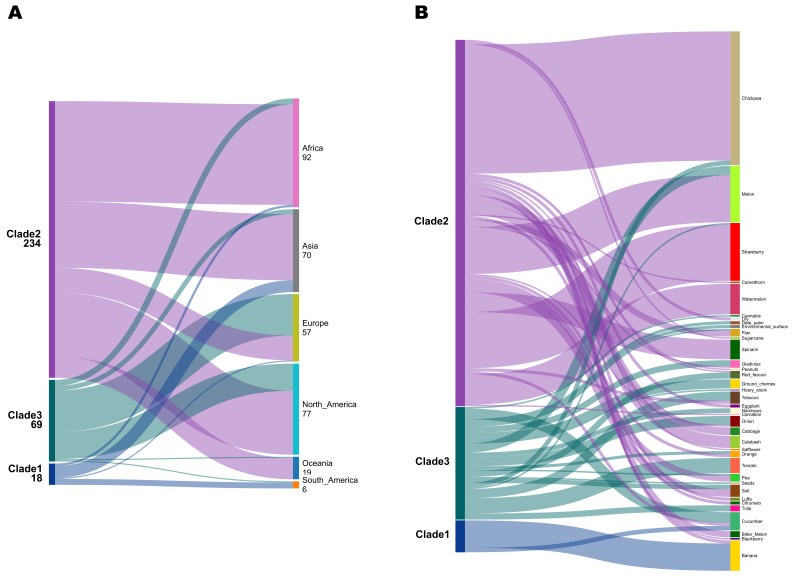
Biogeographic and host distribution tracking of FOSC genomes. Sankey flow diagram mapping the hierarchical epidemiological metadata of the curated dataset. The left panel shows the core-phylogenomic clade assignments defined in this study. Stream (**A**) projects these clades against global geographic distribution across continents (left panel), while stream (**B**) links them to their documented plant hosts or environmental sources (right panel). Genomes with missing, incomplete, or ambiguous metadata records were programmatically excluded to optimize plot interpretability.

High-resolution phylogenomics revealed substantial heterogeneity in the geographic range of the major clades within the FOSC. Clade 2, the largest and most taxonomically diverse clade, exhibited a clearly cosmopolitan distribution. These counts reflect only genomes with available metadata on geographic origin; additional genomes lacking isolation data (NA) are listed separately in the Supplementary Material. The most abundant species were *F. fabacearum* (*n* = 136), predominantly from Africa but also represented in Asia, Europe, and North America, and *F. cugenangense* (*n* = 82), distributed across Asia, Oceania, North America, and Europe. *F. callistephi* (*n* = 6), distributed across Asia, Oceania, and Europe, was intercontinental but less represented, while *F. duoseptatum* (*n* = 4; Asia) and *F. elaeidis* (*n* = 1; Europe) were restricted to single regions. In contrast, *F. libertatis* (Oceania), *F. hoodiae* (Europe, Oceania), and *Fusarium* “afroindicum” (*n* = 4; Africa and Asia) and *Fusarium* “europaeum” (*n* = 4; Europe), not linked to any verified type strain genome in our dataset, showed geographically narrow distributions. Clade 3 displayed a similarly complex structure: *F. nirenbergiae* (*n* = 36) was abundant across Europe, Asia, and North America; *F. curvatum* (*n* = 9) and *F. languescens* (*n* = 10) were intercontinental, spanning both Old and New Worlds; while *F. oxysporum* s. str. (*n* = 8; Europe and Africa) and *F. triseptatum* (*n* = 2; Europe) were confined to the Old World. Other clade 3 members were highly localized, including *F. pharetrum* (*n* = 1; Oceania) and *F. veterinarium* (*n* = 3; North America). Clade 1 consisted solely of *F. odoratissimum* (*n* = 18), which was the most broadly distributed lineage, detected in Asia, Africa, Europe, and South America. Finally, there was *F. foetens* (*n* = 2), detected only in Europe. For several of these species with very low representation (e.g., *F. elaeidis*, *F. duoseptatum*, *F. triseptatum*, *F. pharetrum*, *F. veterinarium*, and *F. foetens*), the apparent geographic and host restrictions of these taxa should be interpreted as preliminary observations conditioned by the limited number of available genomes, rather than as definitive biogeographic or ecological patterns. These contrasting observations of cosmopolitan versus narrowly distributed taxa highlight the uneven evolutionary trajectories within the FOSC, where some lineages have diversified and dispersed globally, while others seem to remain restricted to specific regions or cropping systems (Figure 8).

Differences in host associations were observed both between clades and among the species within them. Within clade 2, the widest spectrum was found in *F. fabacearum*, which encompassed 14 crops spanning at least eight botanical families (Apiaceae, Rosaceae, Asteraceae, Cucurbitaceae, Fabaceae, Rutaceae, Linaceae, Solanaceae). *F. cugenangense* also displayed a broad range, affecting 10 crops such as strawberry, blackberry, melon, watermelon, bitter melon, calabash, lily, spinach, sugarcane, and tobacco. By contrast, other clade 2 members showed narrower associations: *F. callistephi* was restricted to cabbage and pea; *F. duoseptatum* to calabash and luffa; *Fusarium* “europaeum” to flax and onion; and *Fusarium* “afroindicum” to banana and chickpea. *F. elaeidis* and *F. hoodiae* were reported from red fescue and gladiolus, respectively, but due to the very low representation of this clade, no conclusions can be drawn.

**Figure 9:**
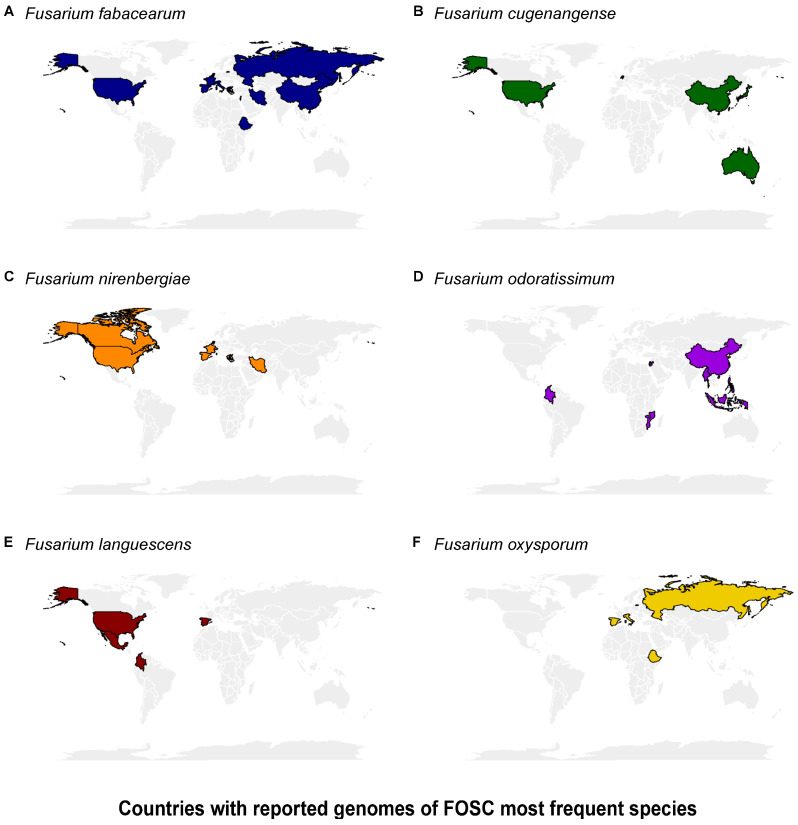
Geographic occurrence patterns of heavily sequenced species within the FOSC. Country-level maps (**A**–**F**) show the documented global distribution of verified genome assemblies for (**A**) *F. fabacearum*, (**B**) *F. cugenangense*, (**C**) *F. nirenbergiae*, (**D**) *F. odoratissimum*, (**E**) *F. languescens*, and (**F**) *F. oxysporum* sensu stricto. Shaded regions identify countries with available genome records in public databases, while gray zones indicate an absence of published data.

In clade 3, the most notable case was *F. nirenbergiae*, associated with 10 crops including strawberry, melon, pea, tomato, sour orange, cucumber, narcissus, onion, gladiolus, and tobacco, spanning seven botanical families. Other clade 3 species exhibited more restricted host profiles: *F. oxysporum* s. str. with chickpea, flax, gladiolus, and red fescue; *F. languescens* with tomato, ground cherries, and melon; and *F. curvatum* with hoary stock, melon, date palm, and tulip. *F. triseptatum* was recorded from red fescue, and *F. veterinarium* from cannabis and environmental surfaces. In clade 1, *F. odoratissimum* was represented by several genomes, the vast majority isolated from banana (Musaceae) and only a few from cucumber (Cucurbitaceae). *Fusarium foetens* is represented only by two genomes, one isolated from begonia (Begoniaceae) and the other from Monterey pine (Pinaceae), indicating a minimal but heterogeneous host record. Restricted host ranges in lineages with very low genomic representation (e.g., *F. elaeidis*, *n* = 1; *F. duoseptatum*, *n* = 4; *Fusarium* “afroindicum”, *n* = 4; *Fusarium* “europaeum”, *n* = 4) should be considered preliminary and contingent upon future sampling efforts. As genomic resources for the FOSC expand, the geographic and host-range characterization of these underrepresented species will likely be refined (Figure 8 and Figure 9).

**Figure 10:**
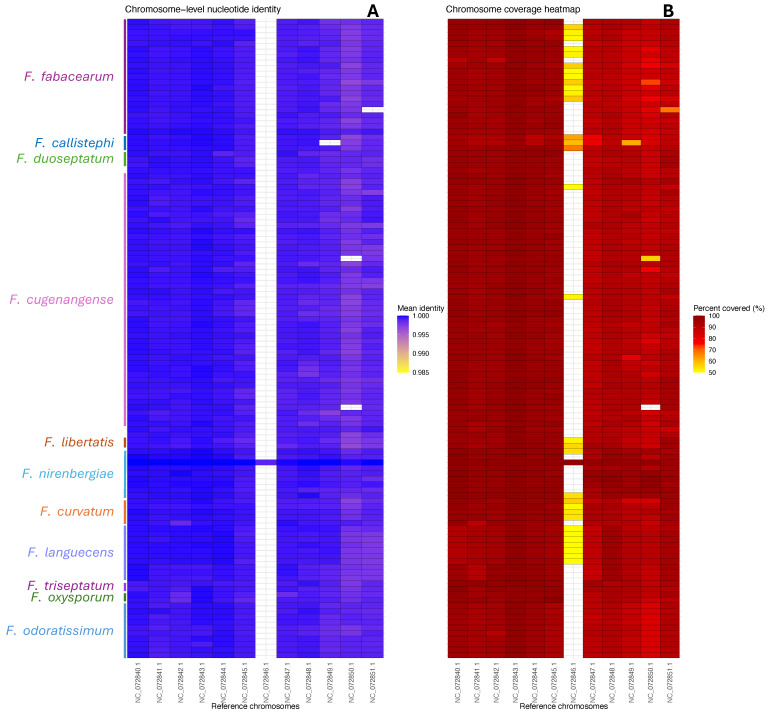
Macro-structural chromosome conservation profiles against reference strain Fo47. Comparative chromosome alignment matrices of FOSC genomes mapped against the structural architecture of *Fusarium oxysporum* reference strain Fo47 (GCF_013085055). (**A**) Heatmap depicting mean nucleotide identity values computed across individual core and accessory reference chromosomes (*x*-axis) against query genomes (*y*-axis). (**B**) Heatmap showing total sequence coverage percentages (%) for the matching genomes, restricted to high-confidence assemblies with ≥100× sequencing depth profiles. Color gradients scale with conservation values; a coverage threshold of ≥50%indicates chromosome presence.

Lastly, we performed genome comparisons at the chromosome level using the reference strain *Fusarium* FOSC Fo47, which is assembled to the chromosome scale, against genomes with high sequencing depth (≥100×). Using a threshold of ≥70% coverage to define chromosome presence, we found that the 11 chromosomes previously described as core were consistently present across strains/species, except for four genomes that showed <70% coverage: two for chromosome NC_072850.1, one for NC_072851.1, and one for NC_072849.1. Nucleotide identity analysis revealed generally high conservation across chromosomes among strains; however, chromosomes NC_072850.1 and NC_072851.1 exhibited the greatest divergence relative to the reference. Notably, NC_072851.1, previously described as specific to Fo47, confirmed this observation. As an alternative approach, we also compared genome coverage of each chromosome using a heatmap-based strategy (Figure 10), this time applying a more relaxed threshold of ≥50%. This analysis revealed slight variations in chromosome coverage across most strains, with some showing <70% coverage for some chromosomes, confirming the previous results. Additionally, this complementary analysis confirms that one strain of *F. cugenangense* had <50% coverage of chromosome NC_072850.1, while the others previously mentioned had coverage between 50–70% for chromosomes NC_072849.1 and NC_072851.1. Interestingly, chromosome NC_072846.1, considered specific to Fo47, appeared to be at least partially present in some FOSC strains when applying the relaxed 50% threshold.

## 3. Discussion

The rapid expansion of global genomic repositories offers an unprecedented opportunity to resolve the complex evolutionary histories of high-impact fungal groups like the *Fusarium oxysporum* species complex (FOSC). However, effectively exploiting these public assets requires navigating substantial heterogeneity in sequencing depth, assembly quality, and strain provenance. Rather than accepting public data at face value, this study underscores the critical necessity of rigorous data curation in fungal macro-evolutionary studies. By implementing a strict, multi-layered quality control framework that excluded over half of the initially available datasets, we established a high-confidence genomic foundation of 336 assemblies. While the broader public database still exhibits baseline challenges—such as assemblies below the ideal 100× coverage threshold or potential indicators of non-clonal sequences—our stringent filtering for N50, BUSCO completeness, and taxonomic purity ensures that the phylogenomic relationships delineated here are highly robust. Moving forward, it is imperative that the mycological community adopts standardized, high-coverage sequencing protocols and transparent metadata reporting to continuously refine the systematic framework established in this work.

Among the recognized species complexes of the genus *Fusarium*, the *F. oxysporum* species complex (FOSC) is consistently highlighted as one of the most relevant groups in agriculture and plant pathology [59], together with the *F. fujikuroi* species complex (FFSC), the *F. graminearum* species complex (FGSC), and the *F. incarnatum*–*equiseti* species complex (FIESC) [43]. However, in its current concept, *F. oxysporum* is a species complex consisting of numerous cryptic species, and the identification and naming of these cryptic taxa are complicated by multiple subspecific classification systems [42]. Moreover, the FOSC encompasses relevant pathogens such as *F. odoratissimum*, *F. oxysporum* sensu stricto, and *F. veterinarium*, whose taxonomic resolution may be limited when relying on conventional mycological approaches. Resolving its evolutionary history and establishing a robust phylogenomic framework to address taxonomic inconsistencies is therefore a long-standing necessity. Such progress will help foster a more reliable understanding of the significance of this complex for agriculture, as well as human and animal health. According to NCBI species records from sequenced isolates within the FOSC, misidentifications are very common, and older literature may therefore provide misleading information on species assignments, including their geographical and host associations. This issue reflects the fact that most *Fusarium* isolates cannot be reliably identified to the species level using only morphological traits, as highlighted by multilocus and molecular studies that have revealed extensive cryptic diversity within the FOSC [59].

The definition of *Fusarium oxysporum* remains broad and ambiguous in classical mycology laboratories [42], as it largely relies on morphological traits and subspecific schemes such as *formae speciales*, races, and vegetative compatibility groups (VCGs). While these systems were historically useful for grouping isolates by host range and virulence spectra, they do not reflect evolutionary relationships [6,12,44]. More than one hundred *formae speciales* have been described [58,59], yet phylogenetic analyses consistently demonstrate their polyphyletic nature. For instance, f. sp. *lycopersici* and f. sp. *radicis-lycopersici* often cluster with unrelated or non-pathogenic strains [26,60,61,62], and similar independent origins have been reported for lineages infecting cucurbits, banana, cotton, celery, and lettuce [63,64,65,66,67]. These inconsistencies underscore the limitations of host-based classifications in capturing evolutionary history [68] and highlight their practical implications for pathogen identification, resistance breeding, and disease management. In this context, Fulton et al. (2021) [69] showed that multilocus sequence analyses were insufficient to resolve the races of *F. oxysporum* f. sp. *niveum* from watermelon, further emphasizing the need for genome-scale approaches that provide more robust comparative frameworks and higher-resolution insights into the genetic diversity of the FOSC.

Our phylogenomic approach, based on 4286 single-copy CDSs, provided high resolution and allowed us to resolve the relationships among currently described species confidently. While consistent with previous studies, our work offers a robust framework by excluding contaminated or low-quality genomes and by incorporating a larger dataset of isolates. This strengthens the current classification of the FOSC and supports the evolutionary relationships among its recognized species.

Resolving the evolutionary history of the FOSC also provides valuable insights into its genome evolution. Core features such as genome size, GC content, and gene duplications highlight the highly dynamic nature of these genomes, in agreement with previous reports across the genus [55]. Within the FOSC, distinct differences become apparent: basal clades such as *F. foetens* and *F. odoratissimum* (clade 1) tend to maintain smaller genomes, whereas clades 3 and, most notably, clade 2 exhibit significant genome expansions. The very large genome sizes observed in clade 2 may reflect the accumulation of accessory chromosomes, transposable elements, or horizontal gene acquisitions, which are often linked to host adaptation and pathogenic specialization [70,71]. These patterns suggest that genome expansion within the FOSC is not random but likely linked to the diversification of pathogenic traits and ecological niches. Notably, clade 2 is also the most diverse clade, with the highest number of isolates recorded worldwide across many countries and a wide range of hosts.

Regarding genome conservation, the high nucleotide identity values (above 96%) confirm that the FOSC is, as often portrayed, a compact group of species. Nonetheless, whole-genome analyses provide the necessary resolution to discriminate among its clades and species. Inter-clade and inter-species nucleotide identity decreased by approximately 1%, reaching around 97.5% and 98.4%, respectively, while within-species comparisons in the clade 2 usually showed median identities above 99%. In contrast, the conservation of aligned genome blocks demonstrated a more variable profile, with alignment coverage ranging from about 70% to nearly 100%. This metric should be interpreted with caution, however, as some genomes displayed unusually small assembly sizes that could be related to insufficient sequencing depth. Unlike prokaryotic species delimitation, where a 95% ANI threshold is widely accepted in the literature, no equivalent consensus exists for fungal species complexes. In this study, species boundaries were defined by the convergence of phylogenomic topology and a consistent pattern of nucleotide identity decay across taxonomic levels.

Chromosome-level comparisons further reinforced the distinction between conserved and variable genomic regions within the FOSC. As expected, the 11 chromosomes described as core were largely retained across strains, supporting their essential role in maintaining the genetic integrity of the complex. The few exceptions, where coverage of certain chromosomes fell below the 70% threshold, likely reflect lineage-specific partial chromosomal loss. Importantly, the observation that chromosomes NC_072850.1 and NC_072851.1 displayed the greatest divergence relative to the Fo47 reference highlights potential hotspots of genomic differentiation within the FOSC. The fact that NC_072846.1, previously described as specific to Fo47, was indeed highly divergent and partially absent in most FOSC genomes, further underscores its strain-specific character and possible role in Fo47’s unique ecological adaptations. The relaxed 50% coverage threshold corroborated these findings and additionally suggested that this chromosome, NC_072846.1, may still persist in partial form within other FOSC species. Together, these results, as well as the observed genome size variations within species boundaries, reinforce the concept of a dynamic chromosomal architecture in the FOSC, where core genomic stability coexists with variable, strain-associated elements that may underlie ecological specialization and pathogenic diversity [56].

Within the FOSC, clade 2 represents the largest and most diverse lineage, encompassing seven recognized species and at least two additional undescribed lineages, provisionally designated *Fusarium* “afroindicum” and *Fusarium* “europaeum”. Both behave as independent evolutionary units with strong internal genomic conservation, with inter-species nucleotide identities of 98.02% and 98.05% relative to *F. fabacearum*, consistently below the >99% within-species values observed for recognized FOSC taxa. *Fusarium* “europaeum” displayed higher internal homogeneity than *Fusarium* “afroindicum” (98.61%), suggesting different evolutionary dynamics between the two lineages. Phylogenetic reconstructions further support their distinctiveness: despite short branch lengths within clade 2, the divergence separating *F.* “afroindicum” and *F.* “europaeum” from neighboring taxa exceeds that observed among the closely related *F. fabacearum*, *F. callistephi*, and *F. cugenangense*.

Ecological and geographic patterns reinforce this view. *F.* “afroindicum” has an old-world transcontinental distribution, with isolates recovered from chickpea in Ethiopia and banana in India, highlighting its host range and ecological versatility. By contrast, *F.* “europaeum” appears geographically restricted, found exclusively in Europe on flax (*Linum usitatissimum*) and onion (*Allium cepa*). The convergence of genomic divergence, phylogenetic distinctiveness, contrasting geographic distributions, and distinct host associations supports their provisional recognition as candidate species within the FOSC, pending the availability of additional genomic resources for these lineages. We emphasize that the names *Fusarium* “afroindicum” and *Fusarium* “europaeum” are used here as informal, provisional phylogenomic designations and are not formally erected as new species under the International Code of Nomenclature for algae, fungi, and plants (ICN) in this work; their formal description awaits the availability of preserved type material and complementary phenotypic data.

Our analysis of SRA metadata reveals contrasting global distribution patterns among the most frequent FOSC species. *Fusarium fabacearum*, the most represented species, occurs widely across the Northern Hemisphere, whereas *F. cugenangense* and *F. odoratissimum* display broader, transcontinental ranges, the latter likely linked to the global trade of banana (*Musa acuminata*). In contrast, *F. nirenbergiae* appears confined to the Northern Hemisphere, while *F. languescens* spans the Americas and Europe. Notably, *F. oxysporum* s. str. was only detected in the Old World (Europe, Asia, Africa), a pattern that diverges from earlier literature describing it as a cosmopolitan pathogen across continents and hosts. Although current genomic sampling remains limited, these data suggest that *F. fabacearum*, rather than *F. oxysporum* s. str., may represent the most globally widespread member of the complex and the most successful, given its recovery from a broader range of plant hosts.

While this study represents one of the largest phylogenomic datasets assembled for the FOSC to date, the geographic and host representation of publicly available genomes is inherently uneven. Regions with stronger genomic research infrastructure, particularly Europe, Asia, and parts of Africa, are overrepresented, whereas South America, Central America, and large parts of sub-Saharan Africa remain undersampled. Similarly, host representation is biased toward economically important crops such as banana, strawberry, and tomato, while isolates from wild plants, non-agricultural environments, or clinical sources are scarce. These biases likely influence the observed patterns of geographic distribution and host range reported here, and may underestimate the true diversity of the FOSC at a global scale. Future efforts to expand genomic sampling across underrepresented regions and hosts will be essential to refine the phylogenomic framework established in this study.

These findings have direct agricultural and phytosanitary implications. The high frequency of species misidentifications detected when comparing our phylogenomic assignments with original NCBI annotations highlights the limitations of conventional identification methods and their potential impact on disease management strategies, resistance breeding programs, and quarantine measures. Accurate species-level identification within the FOSC is a prerequisite for reliable epidemiological surveillance and the development of targeted disease control strategies, particularly for broadly distributed and host-diverse species such as *F. fabacearum*.

## 4. Materials and Methods

### 4.1. Genome Data Acquisition

This study employed an integrated workflow combining systematic genome collection, de novo genome assembly, quality assessment, conserved core genome annotation, orthology inference, and phylogenomic dataset construction. On 29 March 2023, the NCBI Sequence Read Archive (SRA) [72] was queried for Illumina whole-genome sequencing (WGS) datasets of *Fusarium oxysporum*, *F. foetens*, *F. veterinarium*, and *F. odoratissimum*. Only paired-end datasets with at least 1 GB of sequencing data were retained. Hi-C libraries were excluded. A total of 721 accessions meeting these criteria were downloaded using the SRA Toolkit v3.0.0, along with their associated metadata. Raw reads were processed to remove adapters, trim low-quality bases (<Q30), discard short reads (<70 bases), and filter out reads with ambiguous bases. Genomes were assembled de novo using SPAdes (v3.15.3) [73] with the following parameters for Illumina paired-end data: spades.py --isolate -t 20 -m 300 -k 77,89.

### 4.2. Assembly Quality Assessment

Assembly quality was evaluated using multiple metrics, including total assembly size, number of scaffolds, scaffold N50, proportion of ambiguous bases (Ns), and GC content, calculated with a custom Python v3.9.5 script. Sequencing depth was estimated by mapping the cleaned read datasets to their respective assemblies and calculating per-base and average depth using SAMtools v1.14 [74]. Genome completeness and duplication indices were assessed with the BUSCO pipeline (v5.2.2) [75]. To evaluate clonality and intra-sample variation, alternate allele counts were obtained by analyzing the BAM files generated with SAMtools and BCFtools v1.14 [76]. To ensure suitability for phylogenomics, assemblies were retained only if they met strict yet flexible thresholds: N50 ≥ 50 kb, genome size between 44–75 Mb, ≤50,000 scaffolds, and a minimum sequencing depth of 10×. The summary of SRA run accessions that passed the filters and were used in this study, along with the GCA genome references and their genomic descriptive statistics, is provided in Supplementary Table S1.

### 4.3. Taxonomic Classification of Raw Sequencing Reads

To obtain an overview of the taxonomic composition of the raw sequencing data and to assess the potential presence of non-target organisms, raw reads from each SRA accession were classified using Kraken2 v2.1.6. Classifications were performed against a comprehensive reference database comprising bacterial, archaeal, viral, fungal, plant, and metazoan genomes, enabling broad detection of possible contaminants. Because k-mer-based classifiers may be sensitive to database composition and read fragmentation, Kraken2 results were interpreted as indicative rather than definitive assignments.

To derive more conservative estimates of taxon abundance, Kraken2 outputs were further processed with Bracken, which re-estimates read counts by accounting for k-mer distributions and genome length. Bracken abundance estimates were then summarized at the genus level using a custom shell script that aggregated read counts across taxonomic ranks. These genus-level summaries were used to estimate the relative contribution of *Fusarium* reads in each dataset and to identify the second most abundant non-*Fusarium* genus, thereby quantifying the proportion of potential non-target taxa (Supplementary Table S1).

### 4.4. BLAST-Based Post-Assembly Filtering of Residual Contamination

To assess and mitigate the potential impact of residual non-*Fusarium* contamination on genome size and GC content estimates, we implemented an additional post-assembly filtering step based on BLASTN (2.12.0+) similarity searches. Assembled contigs were queried against a custom reference database comprising the finished genome of *Fusarium oxysporum* Fo47 (GCF_013085055) and a curated subset of representative FOSC genomes spanning multiple species. These reference genomes were selected based on optimal assembly statistics and minimal contamination signals identified through Kraken-based taxonomic profiling (ERR4080471, ERR1755748, SRR5725024, SRR10313874, SRR14342093, SRR10428564, SRR10428572, SRR10428583, SRR10428586, SRR10428591, SRR10428593, and SRR10428601). BLASTN searches were performed using stringent thresholds (E-value < 1 × 10^−100^ and bit score ≥ 1000), and only contigs meeting these criteria were retained for downstream analyses. Genome assembly size and GC content were recalculated from the filtered assemblies and used for comparative analyses at the species level (see Supplementary Table S1).

### 4.5. Orthology Inference, Single-Copy Gene Identification, and Phylogenomic Analysis

Single-copy conserved orthologs were identified using BUSCO (v5.2.2) [75]. BUSCO-annotated single-copy proteomes were then processed with SonicParanoid v1.3.5 [77], a graph-based orthology inference pipeline, to cross-validate results and remove residual orthology groups with duplicated loci. The resulting set of single-copy, non-redundant coding sequences (CDS) served as the basis for phylogenomic analysis. A curated set of 4286 single-copy, non-redundant CDS was used for phylogenomic reconstruction. Each CDS was individually aligned using MAFFT v7.490 [78], and alignments were concatenated into a partitioned supermatrix for phylogenetic inference. Phylogenetic inference was performed with IQ-TREE v3 [79] using the best-fit substitution model selected by ModelFinder [80] with a partitioned strategy and the -rcluster 10 option enabled. Branch support was assessed with 5000 ultrafast bootstrap replicates [81]. The resulting tree was rooted with *Fusarium foetens*, and tree topology, branch lengths, and support values were visualized using FigTree v1.4.4 (http://tree.bio.ed.ac.uk/software/figtree/) (Accessed on 1 December 2024). Adobe Illustrator v23 was used for graphical enhancement and coloring of the heat maps.

### 4.6. Genome-to-Genome Comparisons and Nucleotide Identity Analysis

Genome alignments and comparative analyses were performed using the DNADIFF program from MUMMER v3 [82]. All *Fusarium* genomes were aligned pairwise, and the fraction of aligned bases along with the average nucleotide identity were extracted from the “.report” files. A non-redundant summary table was then created and imported into R for downstream analysis.

### 4.7. Genetic Diversity, Host Range, and Metadata Integration

To support phylogenomic reconstruction, metadata on geographic origin and host range were systematically gathered from NCBI BioSample and BioProject records linked to each genome. For each assembly, the collection location (country and continent) and source of isolation, mainly from plants, were recorded. For SRA accessions lacking information on the isolation source or collection site, an extensive search was conducted using BioProject identifiers, strain codes, and related publications to find and add these details to the metadata. Genomes without metadata were labeled as “NA” and kept for comparative analyses.

The curated metadata were integrated with a clade-resolved phylogenomic framework to investigate patterns of diversity, host specialization, and geographic distribution across the three major clades of the FOSC, while also revealing two previously unrecognized lineages within clade 2. To explore and visualize this diversity, we generated Sankey diagrams using SankeyMATIC (https://sankeymatic.com) (Accessed 28 August 2025). These diagrams linked species-level assignments to their corresponding phylogenomic clades, with flow widths scaled proportionally to the number of genomes in each category. In addition, they displayed connections among phylogenomic clades, host types, and sampling continents, thereby integrating taxonomic, ecological, and biogeographic information into a single visualization.

### 4.8. Chromosome Coverage and Identity Against Fusarium Reference Fo47

Pairwise comparisons between a reference genome *Fusarium* FOSC strain Fo47 (GCF_013085055) and a selection of query assemblies filtered by minimum 100× average sequencing depth were performed to assess chromosome-level coverage and sequence identity. Each query assembly was aligned against the reference genome using minimap2 [83] with the asm5 preset, and alignments were filtered to retain only those segments showing at least 95% nucleotide identity. Overlapping alignments were merged with BEDTools v2.30.0 [84], and the cumulative covered bases per chromosome were calculated. Chromosome length information was retrieved from the reference index generated by samtools faidx v 1.14 [74], enabling the computation of the proportion of each chromosome covered by the query assembly. For each chromosome, the mean nucleotide identity was computed as a length-weighted average across all retained alignments. The resulting metrics were compiled into a tabular report containing, for each query-reference comparison, the total aligned bases, reference chromosome length, percentage of coverage, and mean identity.

For visualization, chromosomes were considered present in a given query genome when at least 70% of the chromosome length was covered for chromosome identity analyses, or 50% for chromosome coverage analyses. Two heat maps were generated: the first summarized chromosome presence/absence and mean sequence identity per chromosome using the 70% threshold, while the second represented chromosome coverage, where the presence criterion was relaxed to a minimum of 50% coverage.

### 4.9. Statistical Analyses and Visualization

All statistical analyses and graph generation were performed in R version 4.5.0 [85] with RStudio 2025.05.0+496 [86]. Genome assembly metrics (assembly size, GC content, aligned bases, and average nucleotide identity) were summarized by group using the tidyverse package v2.0.0 [87]. Differences among FOSC clades were evaluated using non-parametric tests: the Kruskal–Wallis test for overall group comparisons and pairwise Wilcoxon rank-sum tests. Boxplots, scatterplots, and comparative visualizations were generated with ggplot2 [88], and statistical annotations were added using ggpubr [89]. Figures were arranged and combined using the ggpubr package v0.6.3.

Geographical distributions of the most frequently represented FOSC species were visualized using world map polygons from the ggplot2 package v4.0.3, with country-level presence annotated from filtered metadata. Countries without reports were shaded light gray, while those with confirmed presence were highlighted. Species maps were combined into a composite panel using ggpubr (ggarrange, annotate_figure) to summarize the global distribution of reported genomes.

## Data Availability

The data presented in this study are openly available in NCBI SRA as listed in Supplementary Table S1.

## Supplementary Materials

The following supporting information can be downloaded at: https://www.mdpi.com/article/10.3390/ijms27146255/s1.

## Abbreviations

The following abbreviations are used in this manuscript:

FOSC	*Fusarium oxysporum* species complex
FFSC	*Fusarium fujikuroi* species complex
FGSC	*Fusarium graminearum* species complex
FIESC	*Fusarium incarnatum*–*equiseti* species complex
WGS	Whole-genome sequencing
SRA	Sequence Read Archive
NCBI	National Center for Biotechnology Information
CDS	Coding sequence(s)
BUSCO	Benchmarking Universal Single-Copy Orthologs
MLSA	Multilocus sequence analysis
HGT	Horizontal gene transfer
VCG	Vegetative compatibility group
UFB	Ultrafast bootstrap
ANI	Average nucleotide identity
BLAST	Basic Local Alignment Search Tool
ML	Maximum likelihood
