# Supplementary figures and images for: Towards a Phylogenomic Framework for the Fusarium oxysporum Species Complex
Source: Int J Mol Sci. 2026 Jul 14;27(14):6255. doi: 10.3390/ijms27146255 (PMC13409881; doi:10.3390/ijms27146255)

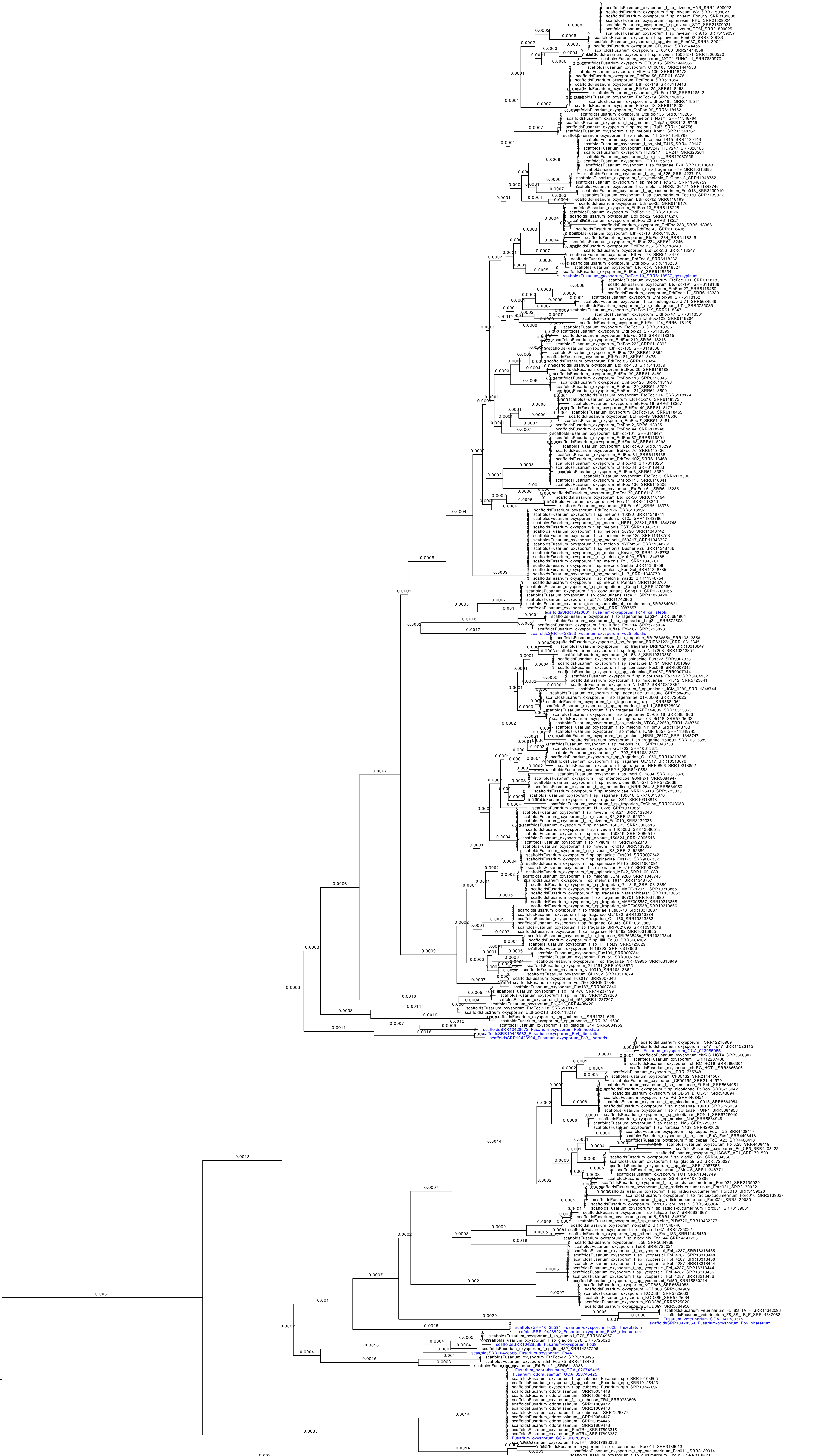

Supplement: Supplementary file 1 [file ijms-27-06255-s001.zip › Supplementary Figure S1.pdf]
